# Supplementary figures and images for: HMGB1 mediates the development of tendinopathy due to mechanical overloading
Source: PLoS One. 2019 Sep 27;14(9):e0222369. doi: 10.1371/journal.pone.0222369 (PMC6764662; doi:10.1371/journal.pone.0222369)

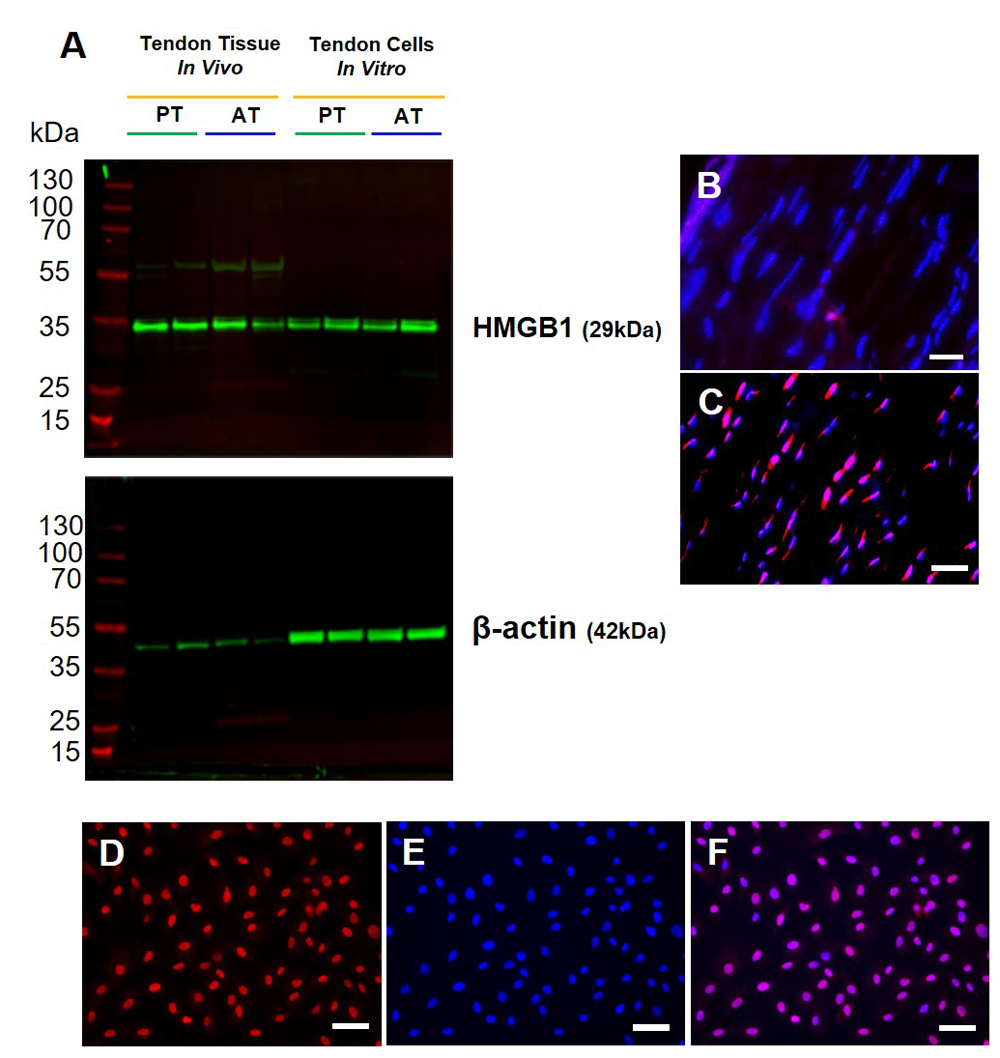

Supplement: S1 Fig — (A) A standard Western blot shows the presence of HMGB1 in both tissues (two samples from different animals) and cells (from two different wells) of patellar tendon (PT) and Achilles tendon (AT). Total protein was extracted from rat Achilles and patellar tendons and cells using T-PER buffer. After quantification, 20 μg of total protein from each tendon sample was separated on a 10% SDS-PAGE, transferred onto a nylon membrane and incubated with rabbit anti-HMGB1 primary antibody (rabbit anti-mouse, 1 μg/ml, Cat # ab18256, Abcam) followed by goat anti-rabbit infrared tag conjugated secondary antibody (1:5,000 dilution, Cat # C30409-07, LI-COR Biosciences, Lincoln, NE) following the manufacturer’s instructions. Positive signals were detected via the Odyssey CLx infrared imaging system (LI-COR Biosciences, Lincoln, NE). β-actin served as internal control. (B) Immunostaining of tendon tissue stained for HMGB1 without penetration with detergent shows that HMGB1 is minimal in tendon matrix. (C) HMGB1 staining in the tendon with Triton X-100 penetration treatment shows that HMGB1 is located in tendon cell nucleus and cytoplasm. (D) HMGB1 staining of tendon cells in culture. Most cells contain HMGB1 in their nuclei (red). (E) Hoechst H33342 stained nuclei (blue). (F) Overlay of both staining (D, E). While HMGB1 is located in the nuclei of most cells (pink), it is missing in some cells. Bar: 50 μm. (TIF) [file pone.0222369.s001.tif]

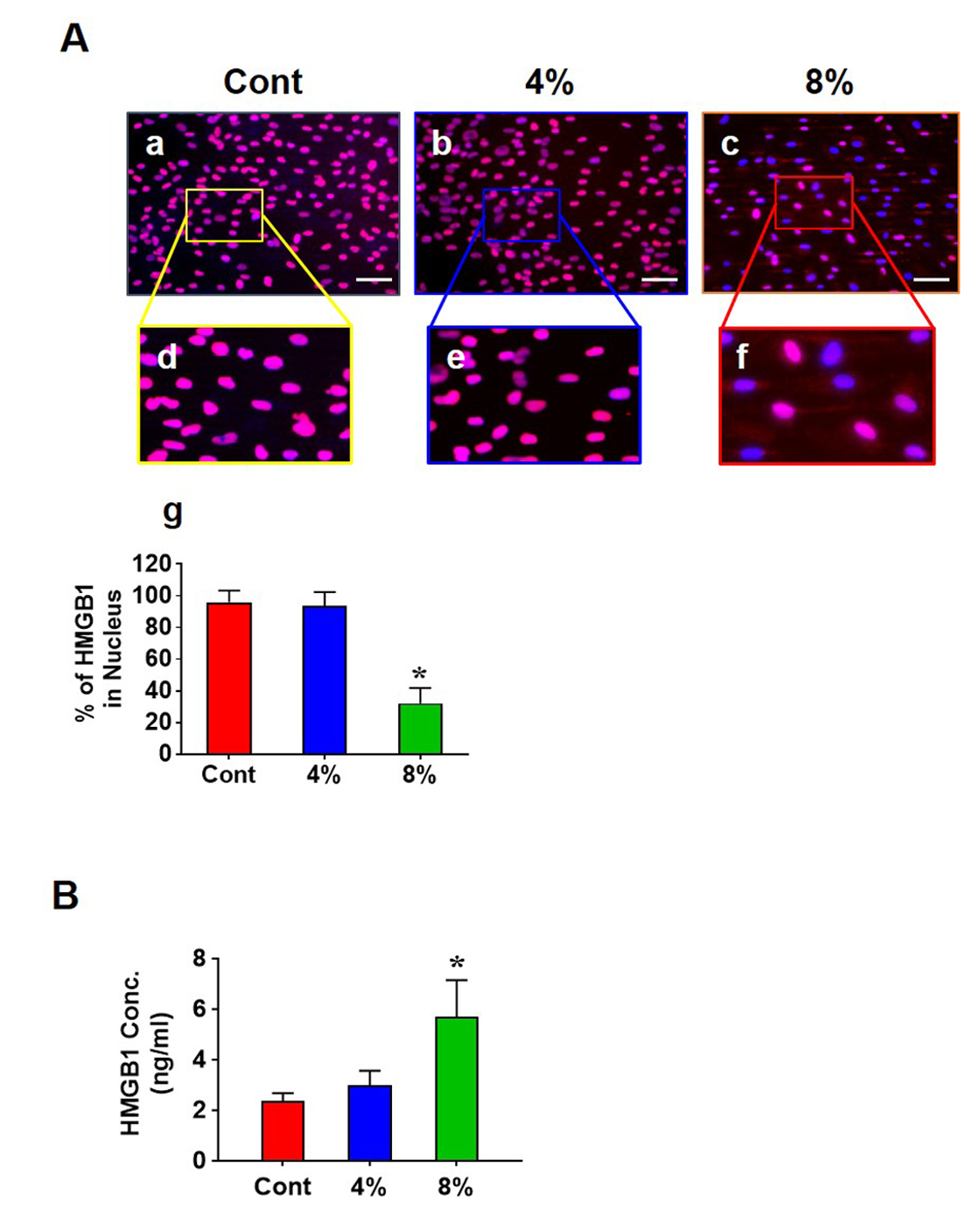

Supplement: S2 Fig — (A) (a, d) Unstretched control cell nuclei stained positive for HMGB1 (pink). (b, e) 4% stretched cell nuclei also stained positive for HMGB1. (c, f) 8% stretched cells show that the majority of cells lose HMGB1 in their nuclei, indicating that their cells have released HMGB1 to culture media under 8% mechanical overloading. Semi-quantification analysis confirms the results (g). Specifically, without mechanical loading or 4% stretching, more than 95% of tendon cells are stained positive for HMGB1. In contrast, there is only about 35% cells that are positive staining with HMGB1, which represents 65% reduction in HMGB1 positive nuclei due to mechanical overloading on the tendon cells. (B) The levels of HMGB1 in culture media were measured using ELISA kits. It is shown that 8% stretch significantly increases HMGB1 levels compared to control and 4% stretch. The cell stretching experiments were done according to our published protocol [15, 60]. All data are means ± SD. n = 6. *P < 0.05. Bar: 50 μm. (TIF) [file pone.0222369.s002.tif]

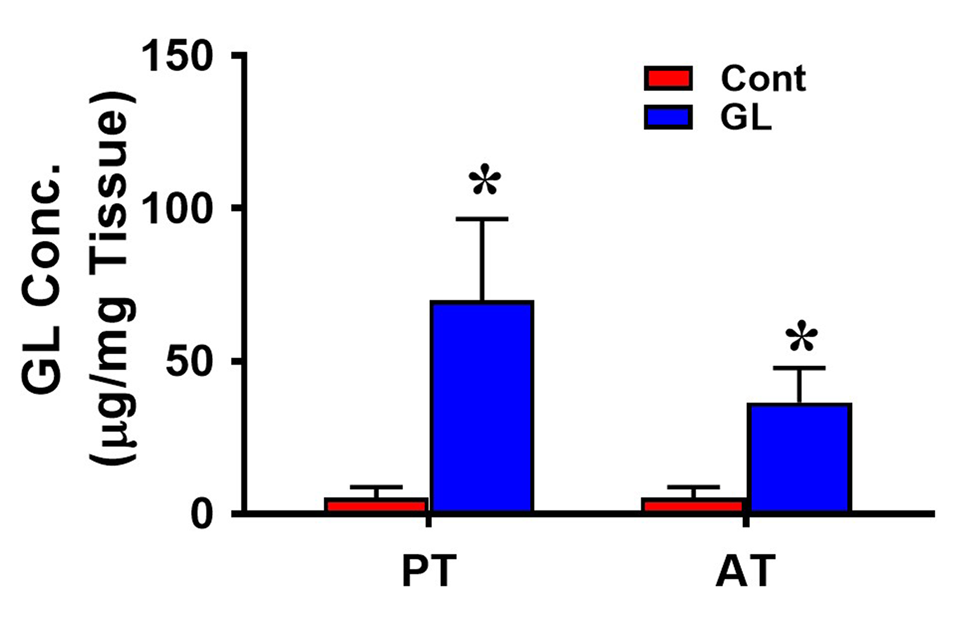

Supplement: S3 Fig — Three hours after IP injection, significant amounts of GL, quantified using thin layer chromatography [61], are detected in mouse tendons. Amount of GL is minimal in mouse tendons without IP injection of GL, but there is 13-fold increase of GL in PT and 6.8-fold increase in AT. PT–patellar tendon, and AT–Achilles tendon. n = 4. *P < 0.05. (TIF) [file pone.0222369.s003.tif]

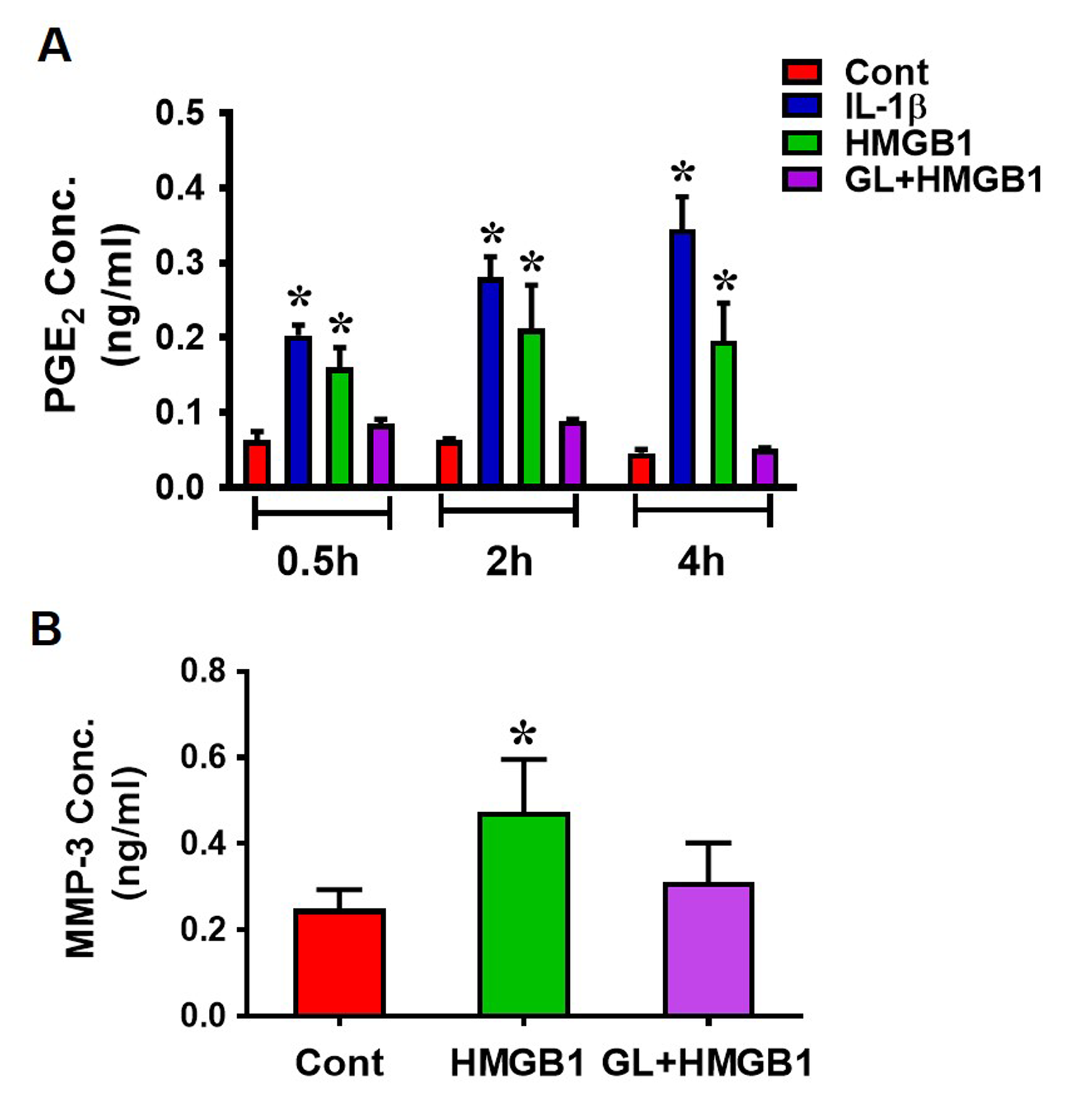

Supplement: S4 Fig — (A) Tendon cells derived from rat Achilles tendons were treated with 10 μg/ml HMGB1 or 10 μg/ml HMGB1+ 200 μM GL in culture, 10 ng/ml IL-1β served as a positive control. PGE2 levels determined by ELISA, significantly increase at 10 μg/ml HMGB1 treatment at 0.5, 2, and 4 hrs, and combined treatment with GL (200 μM) mitigates the effects of HMGB1. (B) HMGB1 treatment (10 μg/ml) of tendon cells significantly increase the production of MMP-3 (ELISA quantification) by tendon cells in culture medium, but addition of 200 μM GL with HMGB1 reduces MMP-3 to a similar level as the non-treated control. Data represent mean ± SD. n = 4. *P < 0.05. (TIF) [file pone.0222369.s004.tif]

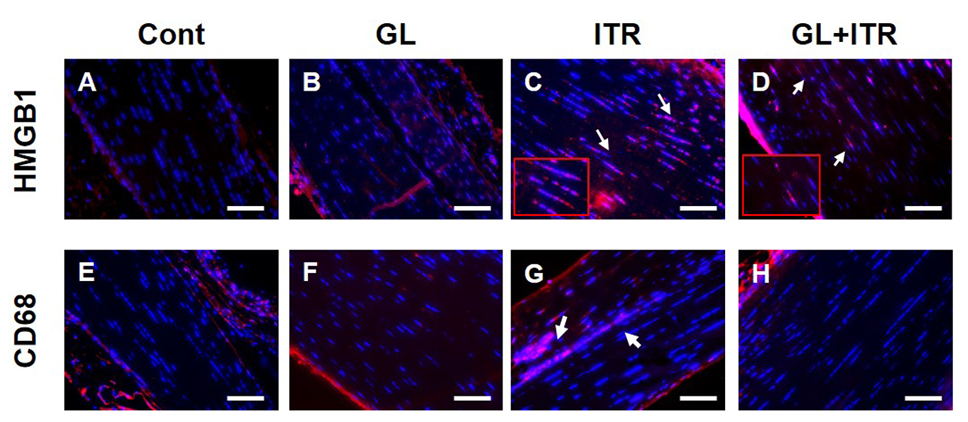

Supplement: S5 Fig — (A, B) In the proximal region of tendinous tissue close to the mouse Achilles tendon-bone insertion site, HMGB1 staining is minimal in control and GL only groups. (C) HMGB1 is present in tendon matrix in the treadmill running group (arrows). (D) HMGB1 is also detected in the tendon matrix of GL+ITR group (arrows). (E, F) CD68 staining is negative in cage control and GL injection only group (yellow arrows). (G) CD68 is positive in ITR group (arrows) and gathered in a clustered form. (H) No positive CD68 signal in the GL-treated ITR tendon tissue. Bar: 50 μm. (TIF) [file pone.0222369.s005.tif]
